# Anaesthetic management of a patient with Eisenmenger syndrome and β-thalassemia major for splenectomy

**DOI:** 10.4103/0019-5049.79892

**Published:** 2011

**Authors:** Nishkarsh Gupta, Sarbjot Kaur, Ajay Goila, Mridula Pawar

**Affiliations:** Department of Anesthesiology and Intensive Care, PGIMER and Associated Dr. RML Hospital, New Delhi, India

**Keywords:** Eisenmenger syndrome, splenectomy, β-thalassemia major

## Abstract

We describe for the first time, the perioperative care of a patient with a rare combination of Eisenmenger syndrome with β-thalassemia major presenting for splenectomy. Patients with Eisenmenger syndrome have polycythemia because of chronic hypoxia but our patient was anaemic and had thrombocytopenia because of thalassemia major. The management of such a case can be challenging for any anaesthesiologist because of severe V/Q mismatch (high shunt fraction and restrictive lung disease because of hypersplenism), decreased oxygen carrying capacity (anaemia) and increased risk of haemorrhage (thrombocytopenia), along with the potential increase in intracardiac shunt during anaesthesia.

## INTRODUCTION

Pulmonary hypertension, with congenital heart disease, is seen in large systemic-to-pulmonary communications, such as ventricular septal defect (VSD) and patent ductus arteriosus. If this is allowed to progress, it leads to irreversible changes in the vessel wall with reversal of shunt and this condition is termed Eisenmenger syndrome. Once Eisenmenger syndrome develops, repair of congenital heart disease is contraindicated.[1]

β-thalassemia majoris an autosomal recessive trait, which causes severe microcytic, hypochromic anaemia, splenomegaly, and severe bone deformities and may lead to death by the second decade. Management consists of periodic blood transfusion; splenectomy if splenomegaly is present, and treatment of transfusion-caused iron overload. We hereby present the anaesthetic management of a rare combination of a case of Eisenmenger syndrome with symptomatic β-thalassemia major, presenting for splenectomy.[2]

## CASE REPORT

A 5-year-old female weighing 12 kg; a known case of Eisenmenger syndrome and β-thalassemia major with hypersplenism presented to us for splenectomy.

She had undergone pulmonary artery (PA) banding with clipping for VSD at 4 months of age. She was diagnosed to have Thalassemia major at the age of 6 months and had been receiving transfusions every 2 months. Frequency of transfusions had increased to once per week recently. On examination, she had facial puffiness, cyanosis, clubbing, pedal oedema, a heart rate of 124 beats/min, a blood pressure of 110/60 mmHg, a respiratory rate of 36/min and oxygen saturation (SpO_2_) of 40% on room air. Systemic examination revealed pan systolic murmur of Grade III, clear lung fields, hepatomegaly (8 cm below costal margin), and splenomegaly (20 cm below left costal margin). She had haemoglobin of 8 gm%, total leucocyte count 2300/mm^3^, platelet count 20,000/ mm^3^, and serum bilirubin (total) 2.2mg/dL. The rest of the liver function and kidney function tests were within normal limits.

2-D echocardiography showed VSD, right to left shunt across the VSD, right ventricular hypertrophy with pressure gradient across pulmonary artery of 105 mmHg. Arterial blood gas (ABG) revealed pH of 7.46, pCO_2_ 48.9 mmHg, and pO_2_ 25.8 mmHg.

The patient was on tab. furosemide 10 mg BD and tab. enalapril 1.25 BD. She was planned for splenectomy and had received pneumococcal, meningococcal, typhoid, and influenza vaccination 7 days prior to the planned surgery. She received antibiotics for infective endocarditis (IE) prophylaxis.

Preoperative electro cardiogram (ECG), invasive blood pressure (IBP), SpO_2_, end tidal carbon dioxide (etCO_2_), temperature, and urine output were monitored. Her baseline parameters were BP 108/72 mmHg, heart rate 86 beats/min, central venous pressure (CVP) 8 mmHg, and SpO_2_ 60%, which improved to 80% with oxygen at 4 L/min through a face mask. The child was premedicated with Inj. midazolam 0.5 mg Intravenous (I.V) and Inj. fentanyl 20 µg. Anaesthesia was induced with Inj. ketamine 10 mg I/V and trachea was intubated with uncuffed endotracheal tube no. 4.5. Postinduction, left radial artery and right internal jugular vein were cannulated. Precautions were taken to avoid the risk of systemic air embolism. Anaesthesia was maintained with halothane < 1% in 100% oxygen, intermittent boluses of inj. atracurium (2.5 mg) and fentanyl (5 µg). The ventilator was set to deliver tidal volumes (6 mL/kg) and the respiratory rate to maintain normocarbia (30-35 mmHg). Initially, airway pressure was 20-30 cm H_2_ O and chest compliance was poor, but postsplenectomy chest compliance improved and airway pressure dropped to <20 cm of H_2_ O. The estimated blood loss was 150 mL, which was replaced with 150 mL blood, in addition to 40 mL ringer lactate. Paracetamol suppository 170 mg was given for postoperative analgesia. As the patient was hemodynamically stable throughout the surgery, the neuromuscular blockade was reversed with neostigmine (0.6 mg) and glycopyrrolate (0.2 mg). Postoperative ABG showed a pH 7.29, pCO_2_ 44.6 mmHg, and pO_2_ 63.7 mmHg, SpO_2_ 88.9%. The patient was transferred to Intensive Care Unit for monitoring. On postoperative day 1, patient was maintaining the vitals. ABG showed a pH 7.45, pCO_2_ 38.6 mmHg, pO_2_ 44.4 mmHg, and SO_2_ 83.2%. Hemoglobin improved to 10.0 g%, platelet count increased to 60,000/mm^3^. The patient was discharged 5 days later from the ward.

## DISCUSSION

Eisenmenger syndrome is defined as pulmonary hypertension due to high pulmonary vascular resistance (PVR) with reversed or bidirectional shunt at an aortopulmonary, ventricular, or atrial level. Patients with Eisenmenger syndrome present with cyanosis, develop polycythemia because of hypoxia and are prone to thromboembolic complications.[1,3]

β-thalassemia major is characterized by impaired production of β-globin chains. This imbalance of globin chain synthesis (α > > β) results in intravascular haemolysis, profound anaemia, erythroid hyperplasia, extramedullary haematopoiesis, splenomegaly, severe bone deformities, and death by the second decade.[2] Management primarily consists of periodic blood transfusion; splenectomy if splenomegaly is present, and treatment of transfusion-caused iron overload.[3]

Our patient had a VSD and was treated with PA banding to reduce PA pressures at the age of 3 months. She was subsequently found to have thalassemia major and required repeated transfusions. This might have suppressed extramedullary haematopoiesis and thus prevented skeletal changes. Thalassemia major might have also contributed to delayed pulmonary hypertension (presenting in this case because of chronic hypoxia) along with PA banding, which resulted in the reversal of shunt and development of Eisenmenger syndrome.

Our patient was anaemic and had thrombocytopenia. The anaesthetic management was complicated because of severe V/Q mismatch (high shunt fraction and restrictive lung disease because of hypersplenism), decreased oxygen carrying capacity (severe anemia), and increased risk of haemorrhage (platelet count 20,000/mm^3^).

During anaesthesia the cardiac output should be maintained and factors that decrease systemic vascular resistance (SVR) or increase PVR, such as hypovolaemia, hypoxaemia, hypercarbia, and acidosis should be avoided.[4,5] Keeping the above goals in mind, we used acombination of fentanyl, low-dose of ketamine and halothane as induction agents. Thiopentone, propofol, and isoflurane were avoided because of their marked effect in lowering systemic vascular resistance. Because of the risk of infective endocarditis, appropriate antibiotics were also administered in the perioperative period.

Excessive transpulmonary pressures can decrease pulmonary blood flow and hence increase the right-to-left shunt.[5] Our patient was paralyzed and intermittent positive pressure ventilation with normal tidal volumes, low peak inspiratory flows with minimal peak airway pressures were used to minimize its effect on the pulmonary blood flow.

Use of invasive monitoring is controversial because of the risk of paradoxical embolism and infection.[6] We had used both IBP and CVP monitoring because of severe pulmonary artery hypertension (PAH) along with associated thalassemia major in our patient. The size of the spleen had a restrictive effect on the respiratory mechanics and had contributed to an increase in V/Q mismatch in our patient. Periodic arterial blood gas determinations showed an increase in PO_2_ from 26 to 36 mmHg immediately postinduction, which further improved to 65 mmHg after abdominal incision and delivery of spleen.

Moreover, patients tolerate volume loss poorly and volume overload may increase the right-to-left shunt, or cause right ventricular failure. So a careful intake output balance was maintained with a CVP of 8-10 cm of water. We could not monitor cardiac output (noninvasive) in our patient, as we did not have access to it.

Since our patient had initial low haematocrit, the risk of thromboembolism was not high unlike in Eisenmenger syndrome. The postoperation monitoring is also very important in these patients as they are prone to arrhythmias and majority of deaths occur in early postoperative period. Our patient was shifted to ICU and intense monitoring (including direct arterial pressure, central venous pressure, oxygen saturation, and ABG) was done. Her vital parameters improved in the postoperative period from the preoperative values, and pain was managed adequately with appropriate analgesics. She was shifted to the ward after 2 days and was sent home 5 days later.

## CONCLUSION

Safe anaesthetic management of patients with Eisenmenger syndrome with thalassemia major requires meticulous preparation, experience, and familiarity with all agents to maintain cardiovascular stability and SVR. Adequate planning and implementation of basic principles had helped us in successfully anaesthetizing this complicated case.
